# Characterization of diffractive bifocal intraocular lenses

**DOI:** 10.1038/s41598-023-27521-7

**Published:** 2023-01-17

**Authors:** Damian Mendroch, Stefan Altmeyer, Uwe Oberheide

**Affiliations:** Institute for Applied Optics and Electronics, Cologne University of Applied Sciences, Betzdorfer Str. 2, 50679 Cologne, North Rhine-Westphalia Germany

**Keywords:** Lens diseases, Characterization and analytical techniques, Confocal microscopy, Applied mathematics

## Abstract

Multifocal intraocular lenses incorporate a variety of design considerations, including dimensioning of the base monofocal shape and the diffraction grating. While studying three different lens models, we present a practical approach for mathematical modelling and evaluation of these geometries. Contrary to typical lens measurement methods, non-contact measurements were performed on the Alcon SN6AD1, HumanOptics MS 612 DAY and the AMO ZMA00 lenses using a confocal microscope. Subsequent data processing includes centering, tilting correction, filtering and an algorithmic decomposition into a conic and polynomial part and the diffraction grating. Lastly, evaluation of fitting parameters and grating shape is done to allow for inferences about further optical properties. Results and analysis show the confocal microscope to be a suitable imaging method for lens measurements. The processing of this data enables the reconstruction of the annular diffraction grating over the complete lens diameter. Apodization, near addition and diffraction efficiency characteristics are found utilizing the grating shape. Additionally, near-optical axis curvature, asphericity and higher order polynomials are identified qualitatively from the reconstruction of the monofocal base form. Derived properties also include the lens optical base and addition power. By making use of the surface geometries, as well as the lens’ material and thickness, a full lens model can be created for further studies. In summary, our analytical approach enables the insight to various intraocular lens design decisions. Furthermore, this procedure is suitable for lens model creation for research and simulation.

## Introduction

An ever-growing trend in cataract surgeries and evermore technological advancements in intraocular lens design and surgery standards still did not displace monofocal lenses in the global market^1^. In contrast, the distinct advantage of the multifocal counterpart are multiple focal distances, therefore mostly or completely eliminating the need for optical aids after surgery. However, multifocal intraocular lenses (MIOLs) come with their own drawbacks. These include halos, glaring and additional chromatic aberration, issues that where especially occurrent in their earlier days.

Many methods and design choices try to counteract the disadvantages, as well as enable a pleasant, mostly aberration-free viewing experience. Besides various design decisions already available for monofocal lenses, including surface curvature, asphericity, higher order polynomials and material choice, multifocal lenses add supplementary options: These comprise dimensioning of the addition power, grating structure, diffraction efficiencies, apodization and choice of underlying lens side. Although definite knowledge would be beneficial for understanding and judgement of different MIOL models and families, manufacturers mostly keep the specific dimensioning a trade secret.

This paper presents a method for measuring multifocal intraocular lenses and deriving a mathematical model, from which geometrical and optical properties are derived. The procedure is performed on three different lenses, but can be extended to various types in further research.

Similar work includes characterization of the diffractive properties of multifocal intraocular lenses by Loicq et al.^2^, as well as measurement and decomposition of the monofocal lens base form by Miret et al.^3^. Other papers derive optical properties from known mathematical lens descriptions, as can be found for the diffractive profile in the work of Vega et al.^4^, and for monofocal intraocular lenses in the work of Barbero et al.^5^.

To our knowledge this research work at hand is the only one deriving a full three-dimensional model from measurement data and studying both monofocal base form and diffractive part.

## Materials and methods

All processing is implemented in Python, while utilizing the well-known scientific libraries numpy, scipy and matplotlib. The code is hosted in the following repository: https://github.com/drocheam/miol-reng-tools.

### Lenses

Investigated lenses include three different models from three different manufacturers, two different materials and three different base and addition powers. While different design aspects would be better compared for models with equal powers, varying values and therefore highly different surface shapes enable us to test the characterization procedure more thoroughly. The examined lenses are as follows:

#### Alcon SN6AD1

The examined AcrySof^®^ IQ ReSTOR^®^ SN6AD1 intraocular lens is specified with 13.0 dpt of optical power and $+3.0$ dpt of near addition. It is made of a highly refractive hydrophobic acrylic material with a refraction index of $n=1.55$. The overall shape is symmetrical biconvex and introduces $-0.1\,$µm of spherical aberration. The apodized diffractive structure is located on the anterior aspheric surface^6,7^.

#### HumanOptics MS 612 DAY

Secondly comes the HumanOptics (formerly Dr. Schmidt) MicroSil^®^ 612 DAY intraocular lens with 25.5 dpt optical power and $+3.5$ dpt near addition. It is made of silicone material and is built as biconvex shape with aspheric surfaces, whereas the anterior side holds the diffractive profile^8,9^. Unfortunately, no data on the refraction index was available. Subsequently a value of 1.47 is assumed, which lies within the typical range for silicone.

#### AMO ZMA00

The third lens is an AMO Tecnis^®^ ZMA00 lens with 30.0 dpt optical power and a $+4.0$ dpt addition. It consists of hydrophobic soft acrylic material with a refractive index of $n=1.47$. The lens is built as a pupil independent, fully diffractive multifocal posterior surface and an aspheric surface on the anterior side. $-0.27\,$µm of spherical aberration are introduced by the lens geometry^2,6,10^.

### Measurement

Commonly used methods for lens measurement include interferometry and profilometry setups^11^. Most of these are highly specialized or cost-ineffective. This work utilizes an available $\upmu$surf^®^ custom confocal microscope by NanoFocus AG, Oberhausen, Germany. In contrast to contact profilometry, the resolution is independent of the stylus size, moreover the lens surface can not be damaged by contact. Additionally, the two-dimensional data output allows for subsequent centering, noise removal and surface smoothing.

Measuring the different microscope focus distances on the edge of the optical relevant area of the lens makes it possible to calculate the tilt vector of the surface. The tilt values are then compensated with the help of a micrometer platform, whereas a residual tilt is removed using software processing shown in “Alignment and centering”. The entire height data consists of multiple sub-images, which need to be stitched together to form one continuous height image. But for the stitching to work effectively, we need to enhance the surface’s information content. This is done by introducing artificial surface impurities to the otherwise smooth lens topography. These consist of microscopic graphite particles with a height around $0.5\,$µm, which will be visible as contamination in the measured data. Sufficient graphite contamination enhances the success rate of the shift detection severely.

The measurement consists of a row of 36 images, with each image being 320 $\upmu$m $\times$ 320 $\upmu$m in size. To improve shift detection, the overlap region between images was set to as much as $150\,$µm. In regard to resolution, in the lateral dimension it is quantified to $0.625\,$µm, while the axial resolution of each image is expected to be better than $0.1\,$µm.

With the confocal measurement being a non-contact method, there are no limitations on the lens material. However, all surfaces need to be in a dried condition, since every remaining liquid film will be interpreted as part of the surface. Also, hydrophilic lenses are known to slightly change their shape while drying, but we currently don’t have any empirical data on how severe the impact on measurements and results will be. An important requirement however is that the surface needs to be reflective for the wavelength range of the microscope illumination. Another aspect is the maximum measurable surface slope, which is limited by the numerical aperture of the objective. For higher slopes the light is reflected away from the beam path of the microscope. In our measurement setup the angle range is limited to $\pm 26.6^{\circ }$, which is sufficient for the examined surfaces.

### Image shift detection

The stepper motor of the microscope is responsible for displacement between individual image acquisitions. However, with the motor being an open loop system, there is no precise feedback on its absolute position and consequently the lateral image shift values. The typical image position uncertainty lies in the order of a few micrometers, but adds up over a large amount of images. With the help of overlapping image regions and methods for shift detection, the shift vectors can be estimated.

Contrary to the built-in shift detection method of the microscope, we implemented a different technique that can be fine-tuned to our needs. This algorithm utilizes the Fourier transformation’s shift theorem. Let the height functions in the overlapping region be

1 $$\begin{aligned} \begin{array}{l} h_{1}(x, y) \\ h_{2}(x, y) \approx h_{1}\left( x-s_{x},~y-s_{y}\right) \end{array} \end{aligned}$$

with the assumption of the second image being the first one shifted by a vector $\vec{s} = (s_x, ~s_y)$. *x* and *y* denote the lateral dimensions, while the axial dimension of the microscope is called *h*. Transforming to the Fourier domain yields:

2 $$\begin{aligned} \begin{array}{l} H_{1}\left( f_{x}, f_{y}\right) =\mathscr {F}\left\{ h_{1}(x, y)\right\} \\ H_{2}\left( f_{x}, f_{y}\right) =\mathscr {F}\left\{ h_{2}(x, y)\right\} \approx H_{1}(f_{x}, f_{y}) \cdot e^{-i 2 \pi \left( f_{x} s_{x}+f_{y} s_{y}\right) } \end{array} \end{aligned}$$

The exponential function *Q* is isolated using the ratio

3 $$\begin{aligned} Q ~=~ \frac{H_{2}\left( f_{x}, f_{y}\right) }{H_{1}\left( f_{x}, f_{y}\right) } ~\approx ~e^{-i 2 \pi \left( f_{x}s_x+f_{y}s_y\right) } \end{aligned}$$

Inverse transformation then yields a Dirac pulse $\delta$ at the image shift position.

4 $$\begin{aligned} V ~=~ \mathscr {F}^{-1}\left\{ Q\right\} ~\approx ~ \delta (x-s_x,~y-s_y) \end{aligned}$$

Finding the maximum in *V* obtains the position of the Dirac pulse and therefore the shift vector $\vec{s}$. Parameters for the stated algorithm include the search region and a threshold for the detection of the peak. In practice, Eq. (1) does not always hold true, because of noise and some information only being present in one of the pictures. Furthermore, the algorithm needs sufficient surface details to find a shift vector. In multiple lens measurements this approach identified shift values for more than $90\%$ of all image transitions, the rest being explained by missing graphite particles in those regions.

### Alignment and centering

The lens center and diameter are elementary for the further processing steps. Within the processing script the user specifies the lens center and edge with the help of a surface derivative image, where slight changes, including the grating rings, are distinctly visible. Next, an optimizing algorithm determines the lens tilt by maximizing rotational symmetry around the lens center. Thereafter, the script corrects the tilt by applying these calculated values.

### Profile decomposition

With a radially symmetric surface, a one-dimensional profile is sufficient for describing the whole topography. For that, the processing script converts the two-dimensional surface image into an average, one-dimensional profile reaching from lens center to the outer edge.

Next, the profile is decomposed into multiple parts for analytical modelling: The radially dependent lens profile *z* consists of a conic section part $z_\text {c}$, a polynomial component $z_\text {p}$ and the diffraction grating $z_\text {d}$, as specified by Eq. (5).

5 $$\begin{aligned} z(r) = z_\text {c}(r) + z_\text {p}(r) + z_\text {d}(r) \end{aligned}$$

Fitting of base shape $z_c(r) + z_p(r)$ and grating $z_d(r)$ are performed independently by assuming that the local changes due to $z_d$ don’t influence the base form consisting of $z_\text {c}$ and $z_\text {p}$. The $z_\text {c}$ component contains an offset $z_0$ and the well-known conic section formula.

6 $$\begin{aligned} z_\text {c}(r)= z_0 + \frac{\rho r^{2}}{1+\sqrt{1-(k+1)(\rho r)^{2}}} \end{aligned}$$

The aim is to determine curvature $\rho$, conic constant *k* and the offset $z_0$ best matching the acquired data. For that reason Eq. (6) is rearranged to:

7 $$\begin{aligned} r^{2}=-z^{2}(k+1)+2 z\left( z_{0}(k+1)+\frac{1}{\rho }\right) -\left( z_{0}^{2}(k+1)+2 \frac{z_{0}}{\rho }\right) \end{aligned}$$

Equation (7) can be represented in the form of a linear equation system $b = Ax$ with:

8 $$\begin{aligned} b=\left[ \begin{array}{c} r_{1}^{2} \\ \vdots \\ r_{i}^{2} \end{array}\right] \quad A=\left[ \begin{array}{ccc} -z_{1}^{2} &{} 2 z_{1} &{} -1 \\ \vdots &{} \vdots &{} \vdots \\ -z_{i}^{2} &{} 2 z_{i} &{} -1 \end{array}\right] \quad x=\left[ \begin{array}{c} k+1 \\ z_{0}(k+1)+\frac{1}{\rho } \\ z_{0}^{2}(k+1)+2 \frac{z_{0}}{\rho } \end{array}\right] \end{aligned}$$

where $r_1 \dots r_i$ and $z_1 \dots z_i$ are the measured values. The overdetermined equation system is best solved using the QR eigenvalue algorithm, which outputs a least-squares fit for *x* at a low computational cost. From *x* the desired values $z_0$, $\rho$ and *k* are found. To minimize the influence of the polynomial component, which is also part of *z*(*r*), only data in the inner $75\%$ of the lens radius is used, where $z_\text {p}$ plays a minor role.

Polynomial regression on the difference $z(r) - z_\text {c}(r)$ with even orders up to $n = 10$ determines the polynomial component $z_\text {p}$. According to Eq. (5) the remaining diffractive part is then $z_\text {d}(r) = z(r) - z_\text {c}(r) - z_\text {p}(r)$. In the last step the diffractive profile is fitted using sectionwise polynomial functions of fourth order.

## Results

### Base shape

Results of the decomposition are shown in Table 1 for the anterior side and in Table 2 for the posterior lens side. *R* and *k* denote the surface curvature and conic constant derived using the fitting in “Profile decomposition”, where *R* is the inverse of the curvature parameter $\rho$. For the sake of simplicity, the height change $h_\text {p}$ due to a polynomial component is listed, instead of specifying individual coefficients. *h* denotes the total height. $d_\text {o}$ is the optical diameter, thus twice the highest radial distance on the surface. Each value is the mean of two independent measurements for every lens side.

**Table 1:** Anterior surface geometry.

Lens	$R_1$ in mm	$k_1$	$h_\text {p1}$ in $\upmu$m	$h_1$ in $\upmu$m	$d_\text {o1}$ in mm
SN6AD1	33.48	− 55.99	0.38	118.9	5.92
MS 612	9.97	1.58	17.45	470.1	6.04
ZMA00	6.55	− 2.12	10.19	609.6	5.83

**Table 2:** Posterior surface geometry.

Lens	$R_2$ in mm	$k_2$	$h_\text {p2}$ in $\upmu$m	$h_2$ in $\upmu$m	$d_\text {o2}$ in mm
SN6AD1	− 32.48	− 8.79	2.27	133.5	6.01
MS 612	− 10.96	− 0.93	5.39	410.2	6.03
ZMA00	− 14.52	− 3.68	− 15.17	310.7	5.94

**Figure 1:**
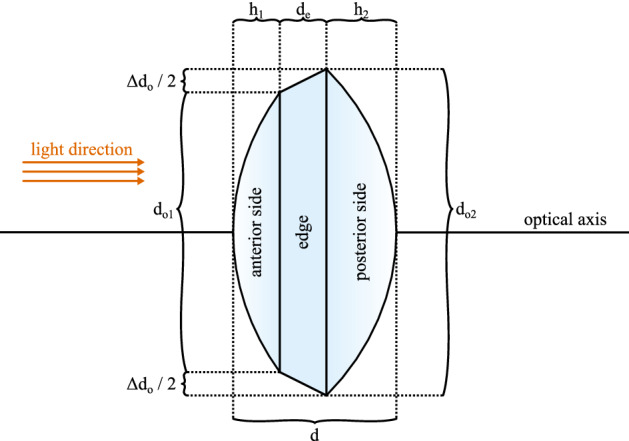
Illustration of lens geometry quantities.

Table 3 shows the resulting properties of the lens with *n* being the refractive index known from manufacturer data, $d_\text {e}$ the size of the lens edge, $\Delta d_\text {o}$ being the difference in optical diameter of back and front side. The overall thickness *d* is the sum of *h* of both lens sides as well as the edge thickness $d_\text {e}$. $d_\text {e}$ was determined with an additional confocal measurement of the lens edge. *D* denotes the base power of the lens and $D_\text {add}$ the near addition. A graphical overview of the geometrical quantities is illustrated in Fig. 1.

**Table 3:** Lens properties.

Lens	*n*	$d_\text {e}$ in mm	$\Delta d_\text {o}$ in mm	*d* in mm	*D* in dpt	$D_\text {add}$ in dpt
SN6AD1	1.55	0.18	0.09	0.43	12.97	2.94
MS 612	1.47	0.29	− 0.01	1.17	25.53	3.46
ZMA00	1.47	0.20	0.11	1.12	29.54	4.24

The optical base power results from the lens maker Eq. (9) with the aqueous humour index of $n_\text {a} = 1.336$^15^. Further, the near addition power $D_\text {add}$ is the result of calculations described in “Near addition power”.

9 $$\begin{aligned} D = \left( n - n_\text {a}\right) \left( \frac{1}{R_1} - \frac{1}{R_2} + \frac{\left( n - n_\text {a}\right) d}{n R_1 R_2}\right) \end{aligned}$$

Although being declared as symmetrical biconvex, the SN6AD1 shows slightly varying curvature radii $R_1$ and $R_2$, the same being the case for the HumanOptics MS 612. The AMO ZMA00 has strongly varying radii, with a ratio of around 1:2.2 between anterior and posterior side. The Alcon SN6AD1 and AMO ZMA00 show a conic constant $k < -1$ on both lens sides, resulting in an outwards declining surface curvature and therefore a lower optical power outside the lens center. This is the expected behavior for lenses with negative spherical aberration, as is specified by the data sheets of the lenses. On the other hand, conic constants $k_1$ and $k_2$ varying in sign, like for the MS 612, are an indication for a lens design with no added spherical aberration.

Lenses MS 612 and ZMA00 feature a higher order polynomial component $h_\text {p}$, while the minor polynomial parts for the SN6AD1 front side are probably due to measurement uncertainties and processing artefacts. The thickness *d* of the HumanOptics and AMO lens is similar, whereas the Alcon model has a thin lens design with only $d=0.43\,$mm. This is the result of the lower optical base power, which produces smaller heights $h_1$ and $h_2$.

Another interesting aspect is the optical diameter difference $\Delta d_\text {o}$ in Table 3. The front of the SN6AD1 is smaller by roughly $90\,$µm and for the ZMA00 by $110\,$µm, respectively. This design choice is motivated by minimizing stray light: An angled lens edge reflects impinging light away from the inner parts of the retina, a curved lens edge additionally distributes the light heterogeneously inside the eye^12^. Evidently, Alcon and AMO are known to incorporate such a design in similar lens models and families^13^.

### Diffraction gratings

**Figure 2:**
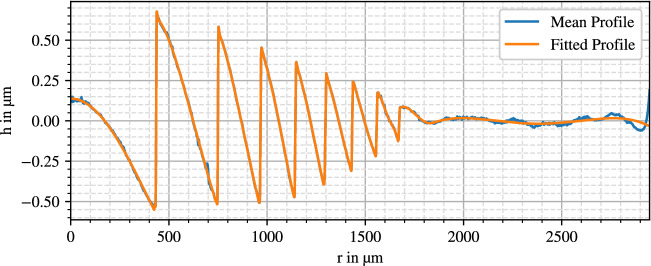
SN6AD1 anterior diffraction profile.

**Figure 3:**
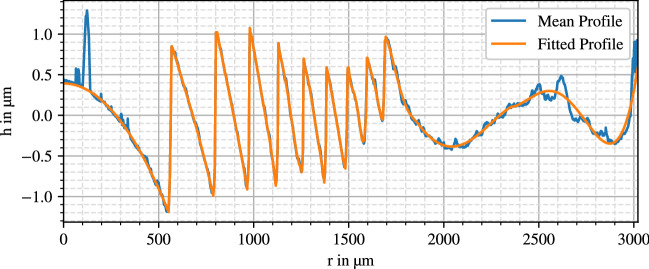
MS612 anterior diffraction profile.

**Figure 4:**
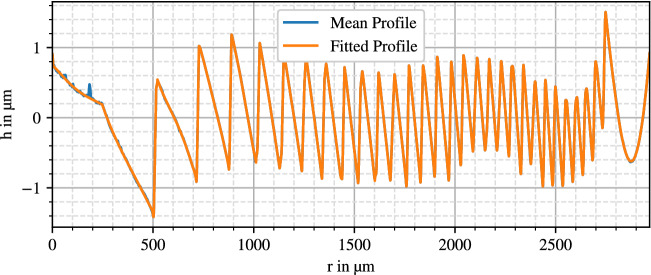
ZMA00 posterior diffraction profile.

Resulting diffraction gratings are displayed in Figs. 2, 3 and 4.

The curves coincide with the expected behavior of a kinoform phase grating. However, the innermost zone of the AMO ZMA00 deviates by consisting of two linear segments. The SN6AD1 grating shows eight steps, the MS 612 nine steps and the ZMA00 lens 29 zone steps. While the diffractive profile follows through the whole surface of the ZMA00, for the two other lenses it only exists at the inner $3.6\,$mm diameter.

The Alcon SN6AD1 shows a distinct decreasing apodization, whereas the zone edges have nearly constant height for the AMO ZMA00. While a varying step height is visible on the HumanOptics MS 612, part of it could be a visualization artifact as discussed later in “Design parameter deviation”.

In the lenses’ mean profile curves there are still some remains of noise and surface contaminations like dust or graphite particles, especially noticeable in the HumanOptics MS 612 anterior profile. Aside from that, the profiles in Figs. 3 and 4 show a superimposed waviness with an amplitude of around 500 nm, which can be a result of stitching errors or polynomial fitting artifacts.

### Near addition power

**Figure 5:**
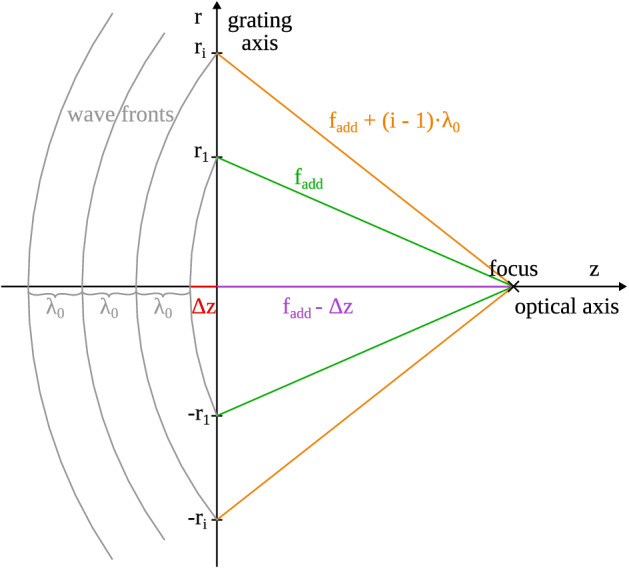
Wave front intersections with the grating axis.

The optical power of a kinoform profile is directly dependent on the annular zone positions $r_i$. Figure 5 illustrates such a kinoform grating with its focus, whereas the spherical wave fronts moving towards the focus are traced back to the grating to find grating intersections. One can see a dependency of $r_i$ on the zone number $i=1, 2, \dots$, the design wavelength $\lambda _0$, the focal distance $f_\text {add}$ as well as the offset $\Delta z$ at the optical axis.

The offset at the optical axis $\Delta z$ can also be described using a phase offset $\phi _0$:

10 $$\begin{aligned} \Delta z = \frac{\phi _0}{2\pi }\lambda _0 \end{aligned}$$

From the right-angled triangle between the origin, position $r_i$ and the focus follows:

11 $$\begin{aligned} \left( f_\text {add} - \frac{\phi _0}{2\pi }\lambda _0\right) ^2 + r^2_i = \left( f_\text {add} + \left( i - 1\right) \lambda _0 \right) ^2 \end{aligned}$$

Solving for $r^2_i$ yields Eq. (12).

12 $$\begin{aligned} r^2_i = 2 \lambda _0 \; f_\text {add} \left( \frac{\phi _0}{2\pi } + i - 1 \right) + \lambda ^2_0 \left( \frac{\phi _0}{2\pi }\right) ^2 + \lambda ^2_0 \left( i - 1\right) ^2 \end{aligned}$$

With $\phi _0 \in [0, 2\pi )$ the ratio $\frac{\phi _0}{2\pi }$ is bound to [0, 1). Also, for a typical lens $f_\text {add} \gg \lambda _0$ and $f_\text {add} \gg \lambda _0 \left( i - 1\right) ^2$ can be assumed. This simplifies Eq. (12) to:

13 $$\begin{aligned} r^2_i = 2 \lambda _0 \; f_\text {add} \left( \frac{\phi _0}{2\pi } + i - 1 \right) \end{aligned}$$

$\phi _0$ is commonly set to $2\pi$, resulting in:

14 $$\begin{aligned} r_i = \sqrt{ 2 i \lambda _0 \; f_\text {add}} \end{aligned}$$

This relationship is utilized for the MS 612 and ZMA00 lenses. Alcon on the other hand sets $\phi _0 = \pi$, giving us Eq. (15) for the SN6AD1 lens^4^.

15 $$\begin{aligned} r_i = \sqrt{ \left( 2i-1\right) \lambda _0 \; f_\text {add}} \end{aligned}$$

This varying grating offset is already visible in Fig. 2, where the innermost part at $r=0\,$µm starts at half the zone’s step height, compared to the full height seen for the two other lenses.

**Figure 6:**
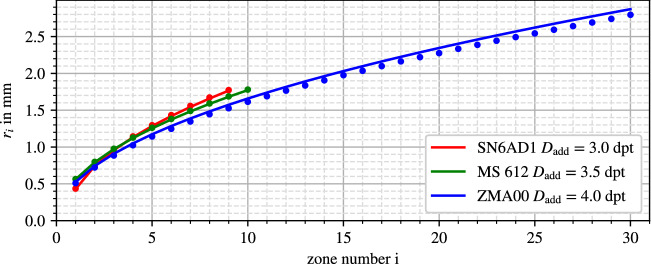
Comparison of measured (dots) and model (line) zone edge positions.

Solving Eqs. (14) or (15) for $f_\text {add}$ makes it possible to determine the power $D_\text {add}$ for each phase zone. Mean values for all zones on each lens are found in Table 3. A comparison of model and measured zone edge locations is displayed in Fig. 6. The lenses SN6AD1 and MS 612 show satisfying compliance with the expected behavior, while the radii of the ZMA00 show a consistent downward deviation.

### Design parameter

The design parameter $\alpha _i$ is defined as^14^:

16 $$\begin{aligned} \alpha _i = \frac{n-n_\text {a}}{\lambda _0}~ h_i \end{aligned}$$

With *n* being the material’s refractive index, $n_\text {a}$ the aqueous refractive index, $\lambda _0 = 550$ nm the design wavelength and $h_i$ the height of the *i*-th zone step. The design parameter directly influences the diffraction efficiency $\eta _j$ for the *j*-th order, as described by Eq. (17)^14^.

17 $$\begin{aligned} \eta _j = \text {sinc}^2(j - \alpha _i) \end{aligned}$$

A value typically chosen is $\alpha _0=0.50$, leading to zeroth and first diffraction order efficiencies being equal at $\eta _{0,1} = 40.5$%.

Design parameter values $\alpha _1$ derived from the innermost zone are depicted in Table 4. Parameters $\alpha _0$ for both SN6AD1 and ZMA00 are known from literature. However, $\alpha _0$ is specified for the center of the lens, deviating from $\alpha _1$ for apodized lenses as the SN6AD1. Nevertheless, the measured values are in proximity to the expected parameters, but show a downward deviation.

**Table 4:** Lens design parameter $\alpha$.

Lens	$\alpha _1$ measured	$\alpha _0$ designed	Reference
SN6AD1	0.47	0.51	^4^
MS 612	0.49	–	–
ZMA00	0.47	0.50	^6^

## Discussion

### Optical power deviation

Compared to manufacturer data in “Lenses”, the optical base powers *D* in Table 3 show small differences. In terms of value this amounts to $0.25\%$ relative error for the SN6AD1, $-0.12\%$ for the MS 612 and the ZMA00 having the highest error with $1.53\%$. All results were within the manufacturing tolerances according to EN ISO 11979-2: 2014^15^. The small metrological deviations are therefore understandable and would have no impact in everyday clinical practice. An overview of designed *D*, $D_\text {add}$ and measured quantities $D_\text {m}$, $D_\text {add,m}$, as well as their relative errors $\Delta D_\text {rel}$, $\Delta D_\text {add,rel}$ can be found in Table 5. According to Eq. (9) deviations in optical power may arise from variations in quantities *n*, *R* or *d*. The curvature radii *R* are expected to be fairly accurate, since the total surface shape contributes to this quantity. Due to a tilted edge and measurement uncertainties of both lens surfaces, we don’t expect the total thickness *d* to be more exact than $\Delta d = \pm 40\,$µm. However, the thickness change $\Delta d$ would account for less than $0.01\,$dpt following Eq. (9) in all investigated lenses. The refractive index *n* is only specified to two decimal places, the index difference $(n - n_\text {a})$ is therefore accurate to merely two significant places. This possibly explains most of the deviation at hand. Nevertheless, for being derived from measured data and simplified considerations, the results provide an excellent fit to manufacturer data.

Relative errors of the near addition $D_\text {add}$ from Table 3 are $1.92\%$ for the SN6AD1, $1.02\%$ for the MS 612 and a value of $-5.94\%$ for the ZMA00. A possible explanation is the location of the diffractive part: While not lying in the principal plane of the lens, its optical power needs to deviate to produce the same effective power. One would expect a lower power value for an anterior diffractive side and a higher value for a posterior one. As it turns out, this exactly matches the deviation’s direction for all three intraocular lenses. However, the discrepancy would be best resolved by simulation of these geometrical models in further research.

**Table 5:** Power deviation comparison.

Lens	*D* in dpt	$D_\text {m}$ in dpt	$D_\text {add}$ in dpt	$D_\text {add,m}$ in dpt	$\Delta D_\text {rel}$	$\Delta D_\text {add,rel}$
SN6AD1	13.00	12.97	3.00	2.94	0.25%	1.92%
MS 612	25.50	25.53	3.50	3.46	− 0.12%	1.02%
ZMA00	30.00	29.54	4.00	4.24	1.53%	− 5.94%

### Design parameter deviation

Differences in the design parameter $\alpha$ in Table 4 are more severe. A change of $\alpha = 0.51$ to $\alpha = 0.47$ produces an absolute efficiency decrease $\Delta \eta _0$ of $-6.5\%$ for the zeroth order. While this form of profile visualization is suitable for a rough estimate, it is not suited for an exact characterization. One possible cause for the deviation is the non-zero width of the zone edges, resulting in a finite edge slope. The grating edges show a typical width of 2–5 $\upmu$m in the radial dimension. This width is based on manufacturing artifacts, although it can be a design decision as well. It is known, that the zone edges result in optical dead zones, regions where rays are refracted a second time, producing stray light and leading to an efficiency loss. By introducing a so-called groove angle, a specifically sloped zone edge, the effect can be compensated^16^. This solution is not only found in Fresnel lenses, but Zeiss is known for incorporating it in the ZEISS AT LARA 829 MP intraocular lens^17^.

The step width leads to a decrease in the step height, since the transition between one zone to another starts at an earlier and ends at a later radial position, disrupting the initial form of the annular zones. Another factor is the converging and pre-focused light coming from the cornea. An angled ray “sees” the projection of the step height, therefore either the height or the slope angle of the steps needs to change to keep the projected height constant. In the latter case an increasing groove angle should be incorporated in the grating design, since the mean incident angle also increases radially. Furthermore, the apodized profile of the SN6AD1 leads to an additionally decreased step height, when measured anywhere except at the lens center.

Finally, some missing height can be explained by the filtering and fitting artifacts from the software processing.

### Comparison to earlier work

Diffraction profile properties of the ZMB00, an MIOL similar to the ZMA00, were already examined by Loicq et al.^2^. But compared to results of our full field measurements only a minor profile section is presented there. Nevertheless, the step heights are showing a similar magnitude. Furthermore, the zone edge positions $r_i$ also show a downward deviation from the model values, although it is less pronounced. However, a lens with base power of $20\,$dpt is measured, while our model has $30\,$dpt. The lens base power has an effect on the lens thickness and therefore the position of the grating relative to the principal plane of the lens. Hence, previous considerations from “Optical power deviation” could explain the higher deviation for this lens.

The surface shape of the monofocal version of the Tecnis^®^, the ZCB00, was studied by Miret et al.^3^. Their work arrives at the conclusion of the anterior side being conic and the posterior side being spherical, both sides without higher order polynomials. These results differ from ours, where both surfaces are aspherical with a polynomial component. It is not obvious from our research, whether the deviation arises from an incorrect surface component decomposition or from the shape of the multifocal version actually being different.

In the case of the Alcon SN6AD1 it is known from Madrid-Costa et al.^18^, that the grating consists of eight steps, therefore nine zones, inside the inner $3.6\,$mm diameter of the lens. Additionally, the grating starts at around half the step height of the first zone. This is in accordance to the results of the work at hand.

Symmetrical, yet slightly different curvature radii of posterior and anterior surface, as well as the highly negative conic constant of the front, are known from manufacturer data of the similar monofocal lens model SN60WF^19^. Unfortunately, no direct comparison is viable, since there is no data provided for a model with the same optical power.

### Data quality

Regarding data quality a differentiation between overall surface shape and derived surface components must be made. For instance, the conic constant *k* is tainted with larger uncertainties, since the profile differs barely from a sphere in the investigated area. The fitting is therefore largely impacted by stitching errors or surface differences mistakenly being attributed to the polynomial component. The same is true for the polynomial part, which proportionally accounts for even a smaller height change. Attribution of surface behavior to an incorrect component does not affect the overall lens shape, since it includes all components altogether. Furthermore, although the superimposed waviness of about $500\,$nm in the diffractive part in Fig. 3 appears large, it is minor compared to the whole height change of $h_1 = 470\,$µm of the surface shape.

Nevertheless, it has to be acknowledged that the decomposition technique from “Profile decomposition” comes with its difficulties. Besides trouble distinguishing surface behavior between the polynomial and conic component, the conic fitting region is a source of result variation. Increasing the fitting region further from $75\%$ of the diameter would lead to slightly different surface components. Another issue is filtering and fitting, due to their low pass properties some abrupt changes in the grating are potentially smoothed out. Further improvements could be made with the usage of a microscope with a precise closed loop stepper motor, eliminating the need for and the errors from the shift detection algorithm.

An alternative asphere fitting approach, including conic section and higher order polynomials, is known from Sun et al.^20^. Their solution is based on an initial linear least squares estimation and an additional non-linear least-squares step. It is not clear, if the mentioned approach would be suited for a surface with an additional grating and grating center height offset like in our application.

Altogether, the data quality is sufficient to identify important properties, as could be seen in the sections before.

## Outlook

The obtained mathematical model should provide an excellent starting point for simulation using established raytracing software like Zemax OpticStudio^®^ or OSLO^®^ from Lambda Research Group. Simulations of these lens models, especially in combination with an adequate eye model, will show how close the model really comes to the expected behavior of the product. The monofocal base shape of the lens can be modelled using the parameters from Tables 1 and 2 and an aspheric surface description in both programs. In contrast, our script exports the whole shape including the grating as a spacing and height dataset. While both programs support user defined surfaces, providing details on how to import this data is outside our expertise.

In further research the processing procedure could be extended to support analysis of trifocal or EDoF (Extended Depth of Field) IOL. While there would be no differences in measuring those lenses, the analysis and mathematical modelling require a different approach. This is especially true for surface shapes that have no rotational symmetry or can’t be decomposed into a simple base and differential profile.

Finally, the presented approach could be performed on more up-to-date and clinically relevant multifocal intraocular lenses to allow for detailed insight into the current lens designs.

## Data Availability

Implemented python scripts as well as a sample measurement data set can be found in the following repository: https://github.com/drocheam/miol-reng-tools.
